# Data describing the solution structure of the WW3* domain from human Nedd4-1

**DOI:** 10.1016/j.dib.2016.06.024

**Published:** 2016-06-22

**Authors:** Vineet Panwalkar, Marianne Schulte, Justin Lecher, Matthias Stoldt, Dieter Willbold, Andrew J Dingley

**Affiliations:** a ICS-6 Strukturbiochemie, Forschungszentrum Jülich, 52425 Jülich, Germany; b Institut für Physikalische Biologie, Heinrich-Heine-Universität, 40225 Düsseldorf, Germany

**Keywords:** Chemical shift, Neuronal precursor cell expressed developmentally down-regulated gene 4-1, NMR, NOE distance restraints, WW domain

## Abstract

The third WW domain (WW3*) of human Nedd4-1 (Neuronal precursor cell expressed developmentally down-regulated gene 4-1) interacts with the poly-proline (PY) motifs of the human epithelial Na+ channel (hENaC) subunits at micromolar affinity. This data supplements the article (Panwalkar et al., 2015) [1]. We describe the NMR experiments used to solve the solution structure of the WW3* domain. We also present NOE network data for defining the rotameric state of side chains of peptide binding residues, and complement this data with *χ*_1_ dihedral angles derived from ^3^*J* couplings and molecular dynamics simulations data.

**Specifications Table**

**Table:** 

Subject area	Biochemistry, structural biology
More specific subject area	Nuclear magnetic resonance (NMR) spectroscopy
Type of data	Tables, figures
How data was acquired	Heteronuclear multidimensional solution-state NMR spectroscopy and MD simulations from experimental structure.
Data format	Processed, analyzed
Experimental factors	The NMR experiments were performed on samples containing 1.5–1.8 mM WW3* domain (^13^C, ^15^N-labeled) from human Nedd4-1 in 20 mM sodium phosphate buffer (pH 6.5), 50 mM NaCl, 0.1% (w/v) NaN_3_ and 1 mM DSS in a 93%/7% (v/v) H_2_O/D_2_O mixture.
Experimental features	All NMR spectra were acquired at 25 °C on Bruker BioSpin Avance III HD 600 and Varian INOVA 900 spectrometers and data were processed using NMRPipe.
Data source location	ICS-6 (Strukturbiochemie), Forschungszentrum Jülich, Jülich, Germany
Data accessibility	Data are within this article and have been deposited in the RCSB Protein Data Bank (http://www.rcsb.org) under the accession number PDB: 5AHT and in the BioMagResBank (accession code: 25349).

**Value of the data**

• The NOE network defines clearly the side chain orientations of particular ligand-binding residues;
• MD simulations provide atomistic descriptions of conformational fluctuations within the WW3^*^ domain that are not observed in the NMR-derived structure of the domain;
• This data set serves as a reference for future studies involving WW domains.

## 1 Data

We have collected 1592 NOE distance restraints from three-dimensional ^15^N-edited and ^13^C-edited NOESY spectra, which were processed using NMRPipe [2] and analyzed using CcpNMR Analysis [3]. The NOE dataset consists of 390 sequential, 416 intra-residue, 266 medium-range and 256 long-range NOE distance restraints. In addition, 60 dihedral angle restraints and five sidechain *χ*_1_ angle restraints determined from combined ^3^*J*_αβ_ and ^3^*J*_Nβ_ couplings were used for structure calculation. The NOEs were picked manually and assigned in a semi-automated manner using the Aria 2.3.1 [4] software package. The structure calculation was carried out by a combination of Aria 2.3.1 and CNS version 1.21 [5] using the PARALLHDG force field. The protocol employed by Aria for calculation of the solution structure of the WW3^*^ domain is provided as supplementary material. The experiments performed to acquire chemical shift assignments, ^3^*J* couplings and NOE distance restraints are summarized in Table 1. The ^3^*J* couplings and the subsequently determined rotameric state for the WW3* domain are given in Table 2.

**Fig. 1:**
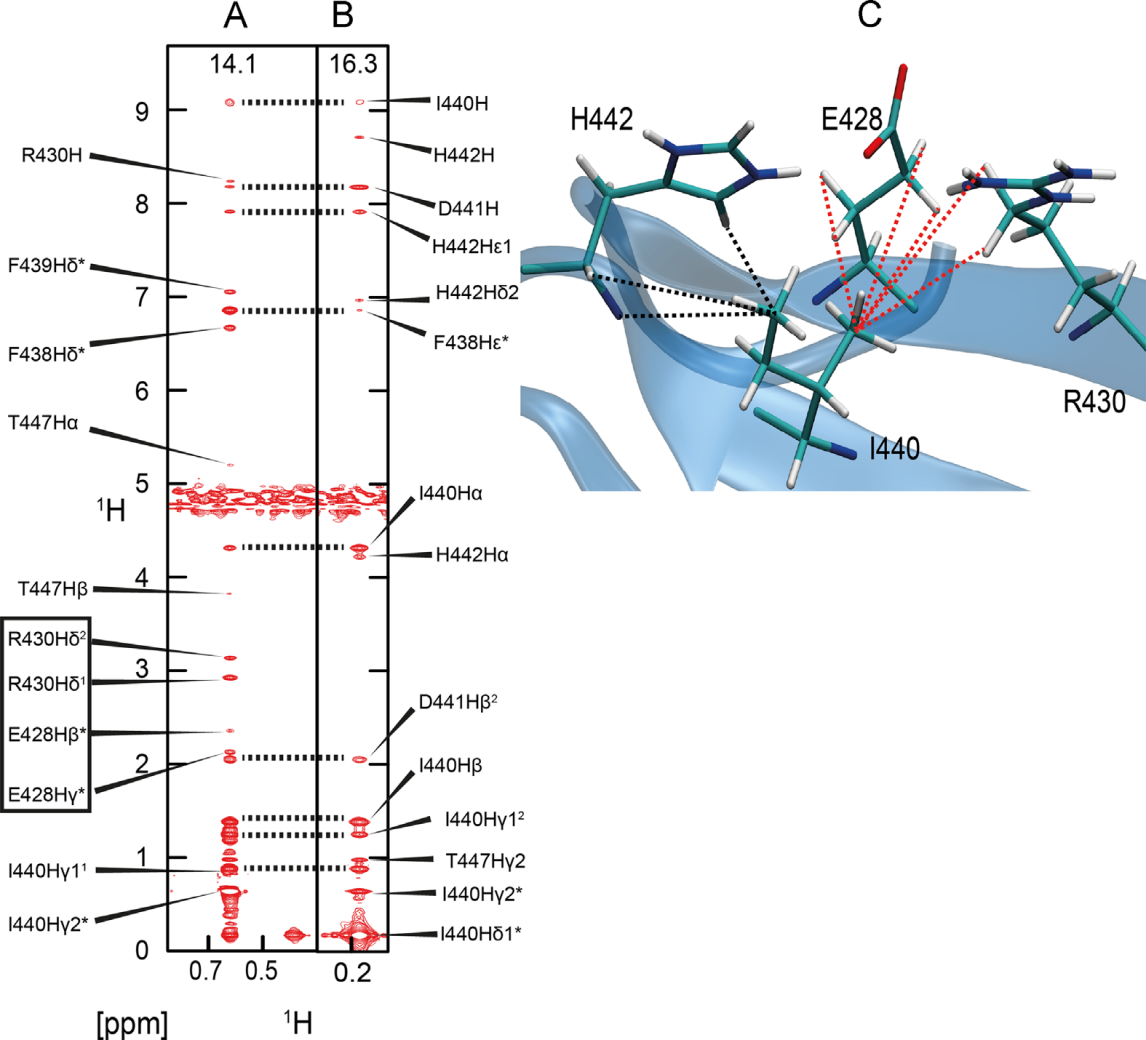
Strips from a ^13^C-edited NOESY spectrum for the δ1 methyl protons (A) and the γ2 methyl protons (B) of the residue I440 of the WW3* domain are shown. The ^13^C chemical shifts are shown at the top of each strip. The NOE network that gives rise to the *trans* rotamer for I440 is mapped onto the structure (C). The γ2 methyl protons show NOEs to the β and γ protons of E428 as well as the δ protons of R430 (red dashed lines in Fig. 1C). The δ1 methyl protons of I440 do not show NOEs to E428 and R430 but show NOEs to the amide proton and the α proton of H442 (black dashed lines in Fig. 1C). This NOE pattern defines the side chain conformation of I440.

**Fig. 2:**
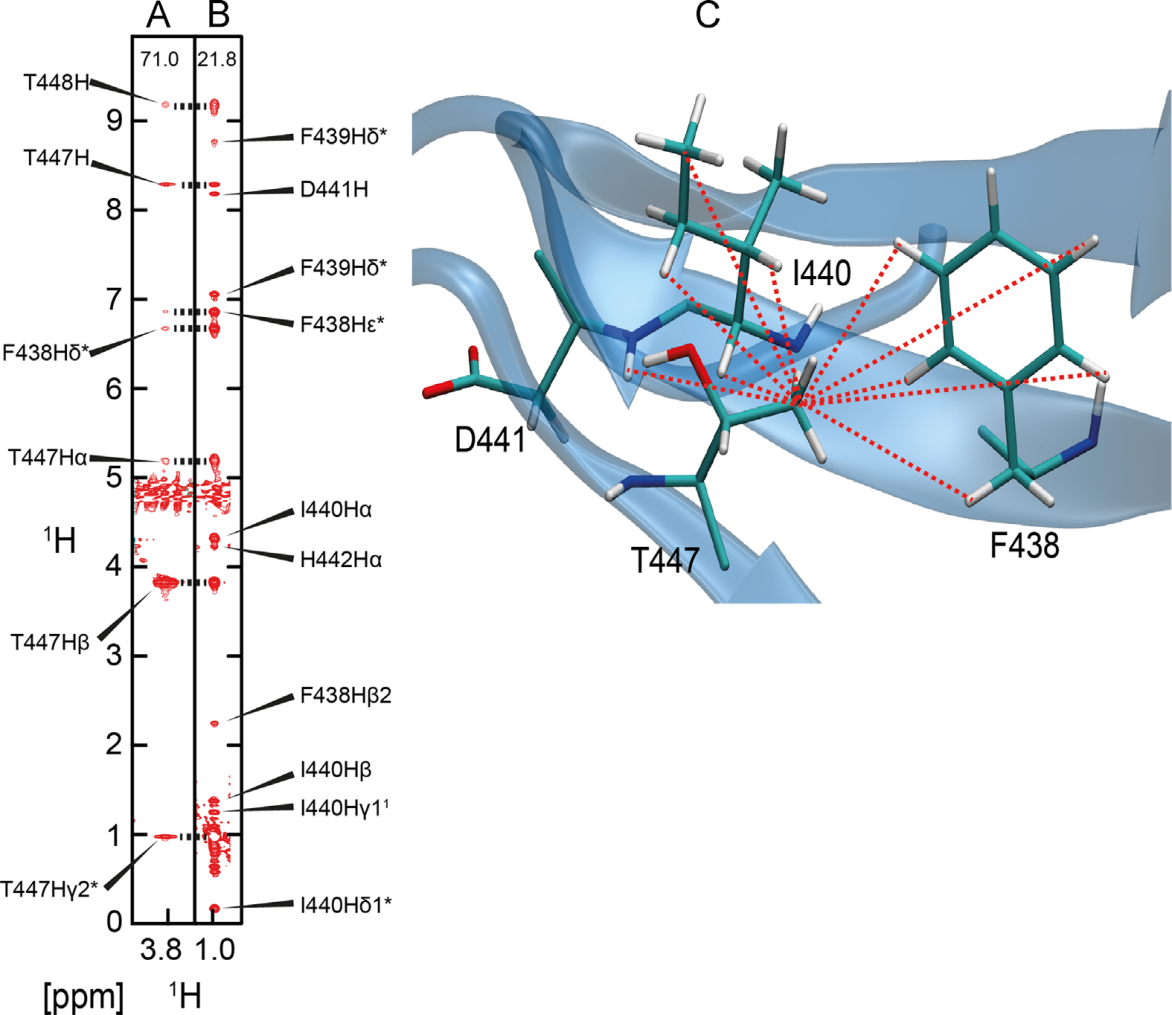
Strips from a ^13^C-edited NOESY spectrum for the β proton (A) and the γ2 methyl protons (B) of the residue T337 of the WW3* domain are shown. The NOE network that gives rise to a *gauche*+ rotamer is mapped onto the structure (C). This NOE pattern defines the side chain conformation of T447.

**Fig. 3:**
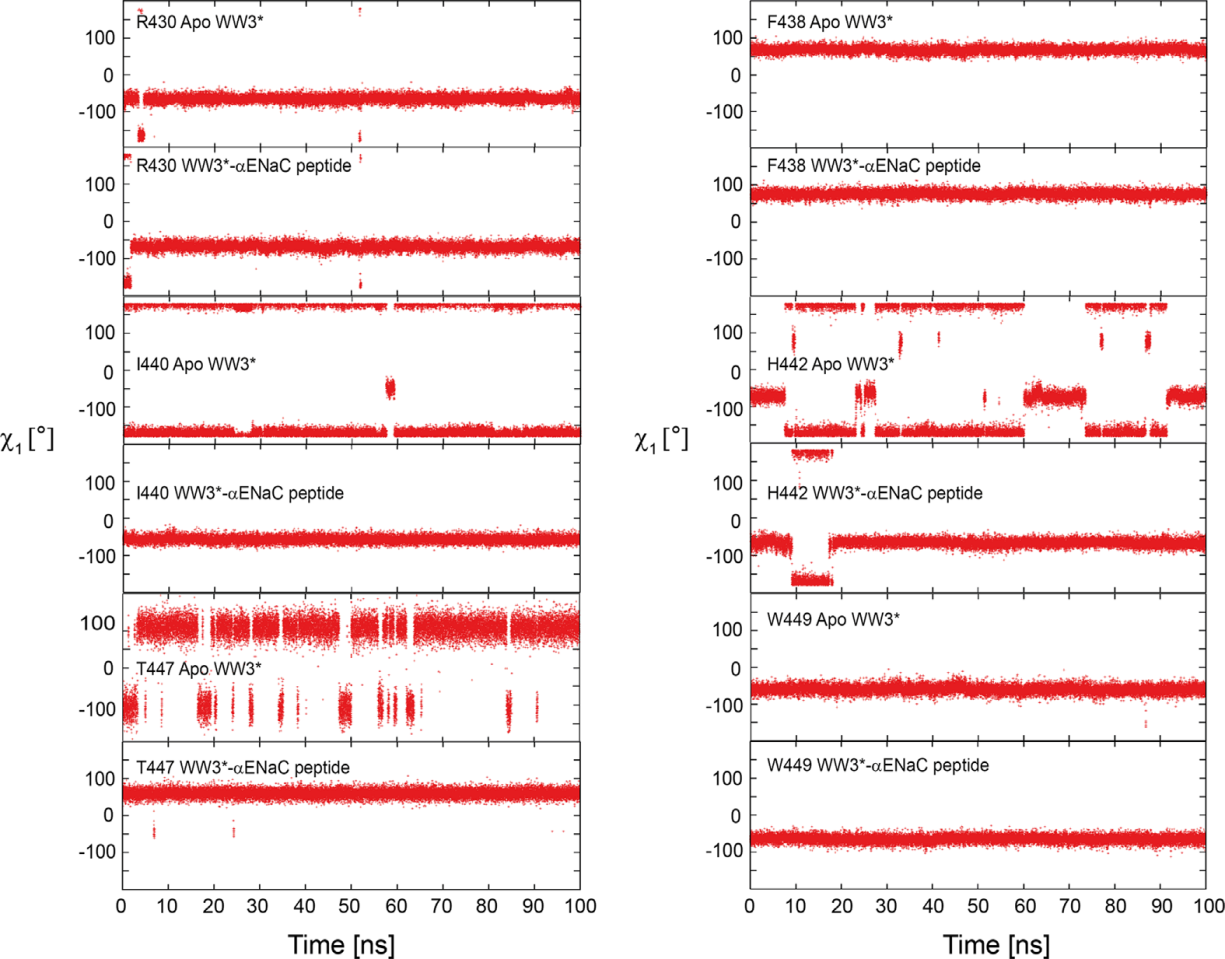
Plots of side chain rotameric states for key peptide binding residues (R430, F438, I440, H442, T447 and W449) observed over 100 ns MD simulations of the apo- and hENaC peptide bound forms of the WW3* domain are shown.

We provide, as examples, the NOE networks for two key peptide binding residues I440 and T447 (Fig. 1, Fig. 2), side chain rotamers of which differ between NMR and the crystal structures [6]. MD simulations data of χ_1_ rotameric states of six key peptide binding residues (R430, F438, I440, H442, T447 and W449) over 100 ns in the apo and hENaC peptide bound state of the WW3^*^ domain is provided (Fig. 3).

## 2 Experimental design, materials and methods

### 2.1 Protein expression, purification and NMR sample preparation

The WW3^*^ domain (41 residues, 4.8 kDa) from neuronal precursor cell expressed developmentally down-regulated gene 4-1 (Nedd4-1) was overexpressed in *E. coli* BL21 (DE3)pLysS cells, as described previously [7], [8]. Protein purification was performed as described previously [1], [7], [8].

### 2.2 NMR spectroscopy

**Fig. 4:**
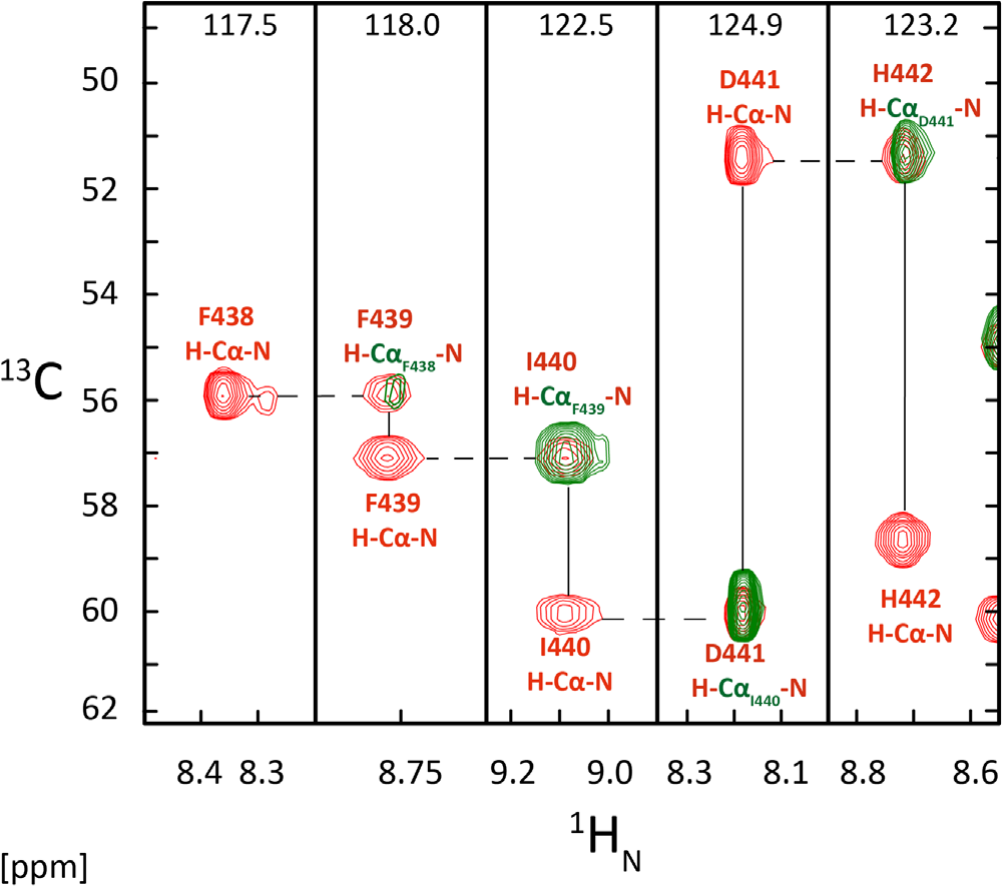
Strips from 3D HNCA (red) and 3D CBCA(CO)NH (green) spectra illustrating the backbone sequential walk from F438 to H442 of the WW3* domain. The ^15^N chemical shift is shown at the top of each strip.

Standard heteronuclear multidimensional NMR experiments [9] were performed on samples containing 1.5–1.8 mM WW3^*^ domain (^13^C, ^15^N-labeled) from human Nedd4-1 in 20 mM sodium phosphate buffer (pH 6.5), 50 mM NaCl, 0.1% (w/v) NaN_3_ and 1 mM DSS in a 93%/7% (v/v) H_2_O/D_2_O mixture. NMR spectra were recorded at 25 °C on NMR spectrometers equipped with cryogenically cooled z-gradient probes operating at ^1^H frequencies of 600 and 900 MHz. ^1^H, ^15^N and ^13^C chemical shift assignments of the WW3* domain were obtained using experiments in Table 1. An example of a backbone sequential walk using three-dimensional (3D) HNCA and CBCA(CO)NH spectra between residues F438 and H442 is presented in Fig. 4. Near complete backbone (193/200 or 96.5%) and side chain assignments (302/319 or 94.5%) were obtained. To derive NOE distance restraints for structure calculation, ^15^N-edited and ^13^C-edited NOESY spectra were recorded using mixing times between 150 and 180 ms. Backbone dihedral angles were obtained from TALOS+ [10] using a combination of backbone (^1^H_N_, ^1^H_α_, ^13^C_α_, ^13^C’ and ^15^N) and ^13^C_β_ chemical shifts. Sidechain *χ*_1_ dihedral angles were obtained from a combination of ^3^*J*_αβ_ and ^3^*J*_Nβ_ couplings derived from 3D HNHB [11] and 3D HAHB(CACO)NH [12] experiments (Table 2).

### 2.3 MD simulations

MD simulations were performed using parameters described in [1].

## Figures and Tables

**Table 1:** NMR experiments used for structure determination.

**Experiment**	**Sweep width (ppm)**	**Data matrices**	**Chemical shift offset (ppm)**	**Number of scans**	**Recycle delay (s)**	**Time (h)**
**Backbone assignments**^a^						
3D HNCO	16 (H)×32 (N)×13 (C)	1024* (H)×32* (N)×40* (C)	4.7 (H)×117.1 (N)×175.2 (C)	8	1.1	15
3D HNCA	12.5 (H)×29 (N)×28 (C)	1024* (H)×42* (N)×64* (C)	4.7 (H)×117.1 (N)×56.8 (C)	8	1.1	28
3D CBCA(CO)NH	16 (H)×32 (N)×50 (C)	1024* (H)×32* (N)×48* (C)	4.7 (H)×117.1 (N)×50 (C)	8	1.1	38

**Side chain assignments**						
3D H(CCO)NH	14 (H)×32 (N)×7.5 (H)	1024* (H)×24* (N)× 64* (C)	4.7 (H)×117.1 (N)×3.0 (H)	16	1.1	38
3D CC(CO)NH	14 (H)×32 (N)×70 (C)	1024* (H)×42* (N)× 64* (C)	4.7 (H)×117.1 (N)×42 (C)	16	1.1	57
3D ^15^N-edited TOCSY	12.5 (H)×32 (N)×12.5 (H)	1024* (H)×20* (N)×50* (H)	4.7 (H)×117.1 (N)×4.7 (H)	16	1.1	24
3D HCCH-TOCSY	6.5 (H)×74 (C)×6.5 (H)	512* (H)×38* (C)×100* (H)	3.2 (H)×45.2 (C)×1.5 (H)	16	1.1	90
2D (HB)CB(CGCD)HD	15 (H)×33 (C)	750* (H)×32* (C)	4.7 (H)×35 (C)	32	1.5	1
2D (HB)CB(CGCDDE)HE	15 (H)×33 (C)	750* (H)×32* (C)	4.7 (H)×35 (C)	32	1.5	1

**Distance restraints**						
3D ^15^N-edited NOESY	15 (H)×27 (N)×12.5 (H)	1024* (H)×46* (N) ×128* (H)	4.7 (H)×119 (N)×4.7 (H)	8	1.2	80
3D ^13^C-edited NOESY	14 (H)×38 (C)×6 (H)	768* (H)×94* (C)×73* (H)	4.7 (H)×29 (C)×2.8 (H)	16	1.1	161
3D ^13^C-edited NOESY(aromatic region)	14 (H)×23 (C)×6 (H)	832* (H)×36* (C)×50* (H)	4.7 (H)×123.4 (C)×7.3 (H)	16	1.1	43

**Dihedral restraints**						
3D HNHB	12.5 (H)×32 (N)×12.5 (H)	1024* (H)×21* (N)×64* (H)	4.7 (H)×117.1 (N)×4.7 (H)	16	1.2	35
3D HAHBCACONH	12.5 (H)×32 (N)×12.5 (H)	1024* (H)×10* (N)×61* (H)	4.7 (H)×117.1 (N)×2.7 (H)	128	1.2	134

a NMR backbone and side chain spectra as well as ^3^*J* data were recorded at 600 MHz, whereas distance restraint experiments were recorded at 900 MHz.

**Table 2:** ^3^*J* couplings and the subsequently derived side chain rotamer used in structure determination of the WW3* domain.

**Residue**	^**3**^***J*****coupling (Hz)**		***χ***_**1**_**angle**
	^**3**^***J*****N**β	^**3**^***J*** αβ	
N434	2.15±0.89, 3.64 ±0.50	3.42±1.02, 4.38 ±0.79	*gauche*-
D441	0.58±0.19, 0.95 ±0.12	N.D., N.D.	*trans*
H442	4.07±0.09, 1.73 ±0.22	3.06^a^, 11.14±1.19	*gauche*+
D451	1.15±0.11, 0.85 ±0.15	N.D., N.D.	*trans*
R453	1.43±0.09, 0.85 ±0.15	4.12 ±1.06, 10.31±0.37	*gauche*+

N.D. Not determined

a upper limit value for the ^3^*J* coupling.
