# Supplementary material for: Data describing the solution structure of the WW3* domain from human Nedd4-1
Source: Data Brief. 2016 Jun 22;8:605–12. doi: 10.1016/j.dib.2016.06.024 (PMC4936499; doi:10.1016/j.dib.2016.06.024)
Supplement: Supplementary file 1 — Transparency document [file mmc1.docx]

*Appendix A: Supplementary material*

**Data describing the solution structure of the WW3* domain from human Nedd4-1**

Vineet Panwalkar^a,^*, Marianne Schulte^a,b^, Justin Lecher^a,b^, Matthias Stoldt^a,b^, Dieter Willbold^a,b^ and Andrew J Dingley^a,^*

*^a^ICS-6 (Strukturbiochemie), Forschungszentrum Jülich, 52425 Jülich, Germany*

*^b^Institut für Physikalische Biologie, Heinrich-Heine-Universität, 40225 Düsseldorf, Germany*

*Address correspondence to either:

Andrew J. Dingley ([a.dingley@fz-juelich.de](mailto:a.dingley@fz-juelich.de)) or Vineet Panwalkar ([v.panwalkar@fz-juelich.de](mailto:v.panwalkar@fz-juelich.de))

*Keywords*: Chemical shift; Neuronal precursor cell expressed developmentally down-regulated gene 4-1; NMR; NOE distance restraints; WW domain

**Protocol employed by Aria for structure calculation of the WW3* domain.**

The protocol can be accessed with Aria as a separate XML file.

<!DOCTYPE project SYSTEM "project1.0.dtd">

<project name="ARIA_NEDD4WW3" version="1.0" author="" date="Tue Sep 24 12:54:50 2013" description="" comment="" references="" working_directory="/home/vpan014/NMR_900_FZJ_data/Aria/changesto_goodone/Stereospecific" temp_root="/home/vpan014/NMR_900_FZJ_data/Aria/tmp" run="17_1" file_root="NEDD4WW3" cache="yes" cleanup="yes">

<data>

<ccpn_model filename="/home/vpan014/NMR_900_FZJ_data/analysis_projects/NEDD4WW3_Stereospecific_7"/>

<molecule file="" format="ccpn" ccpn_id="MS1|A">

<linkage_definition name="automatic" filename=""/>

<parameter_definition name="automatic" filename=""/>

<topology_definition name="automatic" filename=""/>

</molecule>

<spectrum enabled="yes" use_assignments="yes" trust_assigned_peaks="no" structural_rules="no" filter_diagonal_peaks="yes" filter_unassigned_peaks="no">

<shifts file="" format="ccpn" ccpn_id="defaultProject|1" default_shift_error="0.0" use_shift_error="yes"/>

<peaks file="" format="ccpn" ccpn_id="defaultProject|21|1|1" peak_size="volume" freq_window_proton1="0.06" freq_window_hetero1="0.5" freq_window_proton2="0.078" freq_window_hetero2="0.5">

<lower_bound_correction value="0.0" enabled="no"/>

<upper_bound_correction value="6.5" enabled="yes"/>

</peaks>

<experiment_data molecule_correlation_time="0.0" spectrum_mixing_time="0.0" spectrometer_frequency="0.0" ambiguity_type="intra"/>

</spectrum>

<spectrum enabled="yes" use_assignments="yes" trust_assigned_peaks="no" structural_rules="no" filter_diagonal_peaks="yes" filter_unassigned_peaks="no">

<shifts file="" format="ccpn" ccpn_id="defaultProject|1" default_shift_error="0.0" use_shift_error="yes"/>

<peaks file="" format="ccpn" ccpn_id="defaultProject|22|1|1" peak_size="volume" freq_window_proton1="0.03" freq_window_hetero1="0.5" freq_window_proton2="0.09" freq_window_hetero2="0.5">

<lower_bound_correction value="0.0" enabled="no"/>

<upper_bound_correction value="6.5" enabled="yes"/>

</peaks>

<experiment_data molecule_correlation_time="0.0" spectrum_mixing_time="0.0" spectrometer_frequency="0.0" ambiguity_type="intra"/>

</spectrum>

<spectrum enabled="yes" use_assignments="yes" trust_assigned_peaks="no" structural_rules="no" filter_diagonal_peaks="yes" filter_unassigned_peaks="no">

<shifts file="" format="ccpn" ccpn_id="defaultProject|1" default_shift_error="0.0" use_shift_error="yes"/>

<peaks file="" format="ccpn" ccpn_id="defaultProject|25|1|1" peak_size="volume" freq_window_proton1="0.04" freq_window_hetero1="0.56" freq_window_proton2="0.055" freq_window_hetero2="0.5">

<lower_bound_correction value="0.0" enabled="no"/>

<upper_bound_correction value="6.5" enabled="yes"/>

</peaks>

<experiment_data molecule_correlation_time="0.0" spectrum_mixing_time="0.0" spectrometer_frequency="0.0" ambiguity_type="intra"/>

</spectrum>

<dihedrals file="" format="ccpn" ccpn_id="7|1" enabled="yes" data_type="talos"/>

<dihedrals file="" format="ccpn" ccpn_id="50|1" enabled="yes" data_type="standard"/>

<symmetry enabled="no" method="standard" n_monomers="1" symmetry_type="None" ncs_enabled="no" packing_enabled="no"/>

<initial_structure file="" format="iupac" ccpn_id="" enabled="no"/>

</data>

<structure_generation engine="cns">

<cns local_executable="/opt/scisoft/usr/bin/cns-remote" keep_output="yes" keep_restraint_files="yes" create_psf_file="yes" generate_template="yes" nonbonded_parameters="PARALLHDG">

<annealing_parameters>

<unambiguous_restraints first_iteration="0" k_hot="10.0" k_cool1_initial="10.0" k_cool1_final="50.0" k_cool2="50.0"/>

<ambiguous_restraints first_iteration="0" k_hot="10.0" k_cool1_initial="10.0" k_cool1_final="50.0" k_cool2="50.0"/>

<hbond_restraints first_iteration="0" k_hot="10.0" k_cool1_initial="10.0" k_cool1_final="50.0" k_cool2="50.0"/>

<dihedral_restraints k_hot="5.0" k_cool1="25.0" k_cool2="200.0"/>

<karplus_restraints parameter_class="1" a="6.98" b="-1.38" c="1.72" d="-60.0" k_hot="0.0" k_cool1="0.2" k_cool2="1.0"/>

<karplus_restraints parameter_class="2" a="6.98" b="-1.38" c="1.72" d="-60.0" k_hot="0.0" k_cool1="0.2" k_cool2="1.0"/>

<karplus_restraints parameter_class="3" a="6.98" b="-1.38" c="1.72" d="-60.0" k_hot="0.0" k_cool1="0.2" k_cool2="1.0"/>

<karplus_restraints parameter_class="4" a="6.98" b="-1.38" c="1.72" d="-60.0" k_hot="0.0" k_cool1="0.2" k_cool2="1.0"/>

<karplus_restraints parameter_class="5" a="6.98" b="-1.38" c="1.72" d="-60.0" k_hot="0.0" k_cool1="0.2" k_cool2="1.0"/>

<rdc_restraints parameter_class="1" method="SANI" first_iteration="0" k_hot="0.0" k_cool1="0.2" k_cool2="1.0" r="0.4" d="8.0" border_hot_initial="0.1" border_hot_final="40.0" border_cool1_initial="40.0" border_cool1_final="40.0" border_cool2_initial="40.0" border_cool2_final="40.0" center_hot_initial="0.1" center_hot_final="0.1" center_cool1_initial="10.0" center_cool1_final="10.0" center_cool2_initial="10.0" center_cool2_final="10.0"/>

<rdc_restraints parameter_class="2" method="SANI" first_iteration="0" k_hot="0.0" k_cool1="0.2" k_cool2="1.0" r="0.4" d="8.0" border_hot_initial="0.1" border_hot_final="40.0" border_cool1_initial="40.0" border_cool1_final="40.0" border_cool2_initial="40.0" border_cool2_final="40.0" center_hot_initial="0.1" center_hot_final="0.1" center_cool1_initial="10.0" center_cool1_final="10.0" center_cool2_initial="10.0" center_cool2_final="10.0"/>

<rdc_restraints parameter_class="3" method="SANI" first_iteration="0" k_hot="0.0" k_cool1="0.2" k_cool2="1.0" r="0.4" d="8.0" border_hot_initial="0.1" border_hot_final="40.0" border_cool1_initial="40.0" border_cool1_final="40.0" border_cool2_initial="40.0" border_cool2_final="40.0" center_hot_initial="0.1" center_hot_final="0.1" center_cool1_initial="10.0" center_cool1_final="10.0" center_cool2_initial="10.0" center_cool2_final="10.0"/>

<rdc_restraints parameter_class="4" method="SANI" first_iteration="0" k_hot="0.0" k_cool1="0.2" k_cool2="1.0" r="0.4" d="8.0" border_hot_initial="0.1" border_hot_final="40.0" border_cool1_initial="40.0" border_cool1_final="40.0" border_cool2_initial="40.0" border_cool2_final="40.0" center_hot_initial="0.1" center_hot_final="0.1" center_cool1_initial="10.0" center_cool1_final="10.0" center_cool2_initial="10.0" center_cool2_final="10.0"/>

<rdc_restraints parameter_class="5" method="SANI" first_iteration="0" k_hot="0.0" k_cool1="0.2" k_cool2="1.0" r="0.4" d="8.0" border_hot_initial="0.1" border_hot_final="40.0" border_cool1_initial="40.0" border_cool1_final="40.0" border_cool2_initial="40.0" border_cool2_final="40.0" center_hot_initial="0.1" center_hot_final="0.1" center_cool1_initial="10.0" center_cool1_final="10.0" center_cool2_initial="10.0" center_cool2_final="10.0"/>

<flat_bottom_harmonic_wall m_rswitch_hot="0.5" m_rswitch_cool1="0.5" m_rswitch_cool2="0.5" rswitch_hot="0.5" rswitch_cool1="0.5" rswitch_cool2="0.5" m_asymptote_hot="-1.0" m_asymptote_cool1="-1.0" m_asymptote_cool2="-0.1" asymptote_hot="1.0" asymptote_cool1="1.0" asymptote_cool2="0.1"/>

<symmetry_restraints k_packing_hot="15.0" k_packing_cool1="10.0" k_packing_cool2="5.0" last_iteration_packing="8" k_ncs="50.0"/>

<logharmonic_potential enabled="yes" use_auto_weight="yes" weight_unambig="5.0" weight_ambig="0.2" weight_hbond="0.1"/>

</annealing_parameters>

<md_parameters dynamics="torsion" random_seed="89764443" tad_temp_high="10000.0" tad_timestep_factor="9.0" cartesian_temp_high="2000.0" cartesian_first_iteration="0" timestep="0.003" temp_cool1_final="1000.0" temp_cool2_final="50.0" steps_high="10000" steps_refine="4000" steps_cool1="4000" steps_cool2="5000"/>

</cns>

<job_manager default_command="csh -f">

<host enabled="yes" command="qsub -S /opt/scisoft/bin/csh" executable="/opt/scisoft/usr/bin/cns-remote" n_cpu="100" use_absolute_path="yes"/>

</job_manager>

</structure_generation>

<protocol floating_assignment="yes">

<iteration number="0" n_structures="30" sort_criterion="total_energy" n_best_structures="10" n_kept_structures="0">

<assignment/>

<merging method="standard"/>

<calibration relaxation_matrix="no" distance_cutoff="6.0" estimator="ratio_of_averages" error_estimator="distance"/>

<violation_analysis violation_tolerance="1000.0" violation_threshold="0.5"/>

<partial_assignment weight_threshold="1.0" max_contributions="20"/>

<network_anchoring high_residue_threshold="4.0" enabled="yes" min_residue_threshold="1.0" min_atom_threshold="0.25"/>

</iteration>

<iteration number="1" n_structures="30" sort_criterion="total_energy" n_best_structures="10" n_kept_structures="0">

<assignment/>

<merging method="standard"/>

<calibration relaxation_matrix="no" distance_cutoff="6.0" estimator="ratio_of_averages" error_estimator="distance"/>

<violation_analysis violation_tolerance="5.0" violation_threshold="0.5"/>

<partial_assignment weight_threshold="0.9999" max_contributions="20"/>

<network_anchoring high_residue_threshold="4.0" enabled="yes" min_residue_threshold="1.0" min_atom_threshold="0.25"/>

</iteration>

<iteration number="2" n_structures="30" sort_criterion="total_energy" n_best_structures="10" n_kept_structures="0">

<assignment/>

<merging method="standard"/>

<calibration relaxation_matrix="no" distance_cutoff="6.0" estimator="ratio_of_averages" error_estimator="distance"/>

<violation_analysis violation_tolerance="3.0" violation_threshold="0.5"/>

<partial_assignment weight_threshold="0.999" max_contributions="20"/>

<network_anchoring high_residue_threshold="4.0" enabled="yes" min_residue_threshold="1.0" min_atom_threshold="0.25"/>

</iteration>

<iteration number="3" n_structures="30" sort_criterion="total_energy" n_best_structures="10" n_kept_structures="0">

<assignment/>

<merging method="standard"/>

<calibration relaxation_matrix="no" distance_cutoff="6.0" estimator="ratio_of_averages" error_estimator="distance"/>

<violation_analysis violation_tolerance="1.0" violation_threshold="0.5"/>

<partial_assignment weight_threshold="0.99" max_contributions="20"/>

<network_anchoring high_residue_threshold="4.0" enabled="yes" min_residue_threshold="1.0" min_atom_threshold="0.25"/>

</iteration>

<iteration number="4" n_structures="30" sort_criterion="total_energy" n_best_structures="10" n_kept_structures="0">

<assignment/>

<merging method="standard"/>

<calibration relaxation_matrix="no" distance_cutoff="6.0" estimator="ratio_of_averages" error_estimator="distance"/>

<violation_analysis violation_tolerance="1.0" violation_threshold="0.5"/>

<partial_assignment weight_threshold="0.98" max_contributions="20"/>

<network_anchoring high_residue_threshold="4.0" enabled="no" min_residue_threshold="1.0" min_atom_threshold="0.25"/>

</iteration>

<iteration number="5" n_structures="30" sort_criterion="total_energy" n_best_structures="10" n_kept_structures="0">

<assignment/>

<merging method="standard"/>

<calibration relaxation_matrix="no" distance_cutoff="6.0" estimator="ratio_of_averages" error_estimator="distance"/>

<violation_analysis violation_tolerance="1.0" violation_threshold="0.5"/>

<partial_assignment weight_threshold="0.96" max_contributions="20"/>

<network_anchoring high_residue_threshold="4.0" enabled="no" min_residue_threshold="1.0" min_atom_threshold="0.25"/>

</iteration>

<iteration number="6" n_structures="30" sort_criterion="total_energy" n_best_structures="10" n_kept_structures="0">

<assignment/>

<merging method="standard"/>

<calibration relaxation_matrix="no" distance_cutoff="6.0" estimator="ratio_of_averages" error_estimator="distance"/>

<violation_analysis violation_tolerance="0.1" violation_threshold="0.5"/>

<partial_assignment weight_threshold="0.93" max_contributions="20"/>

<network_anchoring high_residue_threshold="4.0" enabled="no" min_residue_threshold="1.0" min_atom_threshold="0.25"/>

</iteration>

<iteration number="7" n_structures="30" sort_criterion="total_energy" n_best_structures="10" n_kept_structures="0">

<assignment/>

<merging method="standard"/>

<calibration relaxation_matrix="no" distance_cutoff="6.0" estimator="ratio_of_averages" error_estimator="distance"/>

<violation_analysis violation_tolerance="0.1" violation_threshold="0.5"/>

<partial_assignment weight_threshold="0.9" max_contributions="20"/>

<network_anchoring high_residue_threshold="4.0" enabled="no" min_residue_threshold="1.0" min_atom_threshold="0.25"/>

</iteration>

<iteration number="8" n_structures="100" sort_criterion="total_energy" n_best_structures="10" n_kept_structures="0">

<assignment/>

<merging method="standard"/>

<calibration relaxation_matrix="no" distance_cutoff="6.0" estimator="ratio_of_averages" error_estimator="distance"/>

<violation_analysis violation_tolerance="0.1" violation_threshold="0.5"/>

<partial_assignment weight_threshold="0.8" max_contributions="20"/>

<network_anchoring high_residue_threshold="4.0" enabled="no" min_residue_threshold="1.0" min_atom_threshold="0.25"/>

</iteration>

<water_refinement solvent="water" n_structures="15" enabled="yes" write_solvent_molecules="no"/>

</protocol>

<analysis>

<structures_analysis enabled="yes"/>

<procheck executable="/opt/scisoft64/usr/bin/procheck" enabled="yes"/>

<prosa executable="/usr/bin/prosa" enabled="yes"/>

<whatif executable="whatif" enabled="no"/>

<clashlist executable="/opt/scisoft64/usr/bin/clashlist" enabled="yes"/>

</analysis>

<report>

<ccpn export_assignments="yes" export_noe_restraint_list="last" export_structures="yes"/>

<molmol enabled="yes"/>

<noe_restraint_list pickle_output="no" text_output="yes" xml_output="no"/>

<spectra write_assigned="no" write_assigned_force="no" iteration="last" write_unambiguous_only="yes"/>

</report>

</project>
