# Supplementary material for: Data describing the solution structure of the WW3* domain from human Nedd4-1
Source: Data Brief. 2016 Jun 22;8:605–12. doi: 10.1016/j.dib.2016.06.024 (PMC4936499; doi:10.1016/j.dib.2016.06.024)
Supplement: Supplementary file 2 — Supplementary material [file mmc2.docx]

Conflict of interests: none
